# A Data-Driven Method to Discriminate Limb Salvage from Other Combat-Related Extremity Trauma

**DOI:** 10.3390/jcm12196357

**Published:** 2023-10-04

**Authors:** Stephen M. Goldman, Susan L. Eskridge, Sarah R. Franco, Jason M. Souza, Scott M. Tintle, Thomas C. Dowd, Joseph Alderete, Benjamin K. Potter, Christopher L. Dearth

**Affiliations:** 1 DoD-VA Extremity Trauma and Amputation Center of Excellence, Bethesda, MD 20889, USA; 2 Department of Surgery, Uniformed Services University of the Health Sciences and Walter Reed National Military Medical Center, Bethesda, MD 20814, USA; 3 Leidos, Reston, VA 20190, USA; 4 Naval Health Research Center, San Diego, CA 92152, USA; 5 Department of Plastic and Reconstructive Surgery, Walter Reed National Military Medical Center, Bethesda, MD 20814, USA; 6 Department of Orthopaedic Surgery, Walter Reed National Military Medical Center, Bethesda, MD 20814, USA; 7 Department of Orthopaedic Surgery, San Antonio Military Medical Center, Houston, TX 78234, USA

**Keywords:** Abbreviated Injury Scale, military medicine, wound and injuries, amputation, musculoskeletal injuries

## Abstract

Introduction: The aim of this study was to address and enhance our ability to study the clinical outcome of limb salvage (LS), a commonly referenced but ill-defined clinical care pathway, by developing a data-driven approach for the identification of LS cases using existing medical code data to identify characteristic diagnoses and procedures, and to use that information to describe a cohort of US Service members (SMs) for further study. Methods: Diagnosis code families and inpatient procedure codes were compiled and analyzed to identify medical codes that are disparately associated with a LS surrogate population of SMs who underwent secondary amputation within a broader cohort of 3390 SMs with lower extremity trauma (AIS > 1). Subsequently, the identified codes were used to define a cohort of all SMs who underwent lower extremity LS which was compared with the opinion of a panel of military trauma surgeons. Results: The data-driven approach identified a population of n = 2018 SMs who underwent LS, representing 59.5% of the combat-related lower extremity (LE) trauma population. Validation analysis revealed 70% agreement between the data-driven approach and gold standard SME panel for the test cases studied. The Kappa statistic (κ = 0.55) indicates a moderate agreement between the data-driven approach and the expert opinion of the SME panel. The sensitivity and specificity were identified as 55.6% (expert range of 51.8–66.7%) and 87% (expert range of 73.9–91.3%), respectively. Conclusions: This approach for identifying LS cases can be utilized to enable future high-throughput retrospective analyses for studying both short- and long-term outcomes of this underserved patient population.

## 1. Introduction

Advancements in military medicine have dramatically improved the survivability of severe combat-related injuries relative to prior conflicts. At least half of the combat-related injuries sustained by US Service members (SM) during the conflicts in Iraq and Afghanistan involved these extremities [1]. Of these traumatic extremity injuries, a preponderance was classified as severe; in other words, documented as serious to fatal based on the Abbreviated Injury Scale (AIS > 1) [2]. Moreover, the severity and complexity of these extremity injuries necessitate an even greater understanding of the acute and long-term care and synergistic efforts among the multidisciplinary team required [3,4] to facilitate the highest possible functional outcome. For example, early in the clinical management of complex extremity trauma, multiple clinical care decisions are made with the goal of maximizing functional outcome potential while minimizing the number and duration of reconstructive surgeries. The most binary of these decisions is whether or not amputation of the affected limb is the best course of care. SMs who do not undergo primary amputation but rather receive extensive surgical and rehabilitative treatments are often referred to as ‘limb salvage’ (LS) cases. While SMs who have undergone LS procedures have traditionally experienced clinical outcomes at or below that of SMs who have undergone amputation [5], there is an understandable preference among the patient and multidisciplinary care teams to perform LS whenever possible. For example, improvements in battlefield care and evacuation, utilization of a patient-centered approach, continual developments in surgical approaches and associated next-generation pro-regenerative technologies, clinical availability of improved orthotic devices, and advanced rehabilitation techniques have all made LS an increasingly viable option [6]. Therefore, it is becoming increasingly important for the clinical and scientific communities to understand the outcomes associated with LS cases in both the short- and long-term. An enhanced understanding of such outcomes will help ascertain the impact of emerging reconstruction techniques, interventions, and rehabilitation methods on both LS and amputation outcomes. This information is critical to (1) allow clinicians and patients to make informed decisions, and (2) enable the scientific community to innovate towards next-generation treatments and devices to further improve outcomes for the LS patient population.

Retrospective analysis of large medical databases represents an important tool for better understanding the clinical outcomes of current treatment modalities used across the medical landscape, including LS procedures. The practical aspects of performing such studies specific to LS have historically been fraught with challenges related to the rather ambiguous nature of how to define LS, especially relative to other more definitive populations (e.g., limb loss). In other words, the boundaries of what types of diagnoses and procedures (and combinations thereof) constitute/define an LS case can be fluid between institutions and providers. Since there are no clearly established criteria for inclusion or exclusion from the LS population, these decisions are often informed by local and/or individual experiences and biases [7]. Subsequently, the literature consists of a number of studies ostensibly pertaining to LS for which the aforementioned confounders may unduly influence the scientific approach and/or outcome. For instance, an authors’ chosen definition may be too restrictive in its definition by placing a requirement of a single injury type (e.g., Gustilo Type IIIB and IIIC, vascular injury) [8,9,10,11,12,13] or it may cast a wide net including a number of more minor injury patterns (e.g., those for which limb loss was never a plausible outcome) [14,15,16]. Regardless, both the specific criteria and the wide net approaches are inherently limited by the a priori selection of inclusion/exclusion criteria based on the authors’ narrow interests and/or preconceived notion of what constitutes a trauma-related LS case. Moreover, conducting retrospective studies in this manner requires a significant human capital investment to perform detailed chart reviews of each member of the study population so as to manually evaluate their status as an LS recipient (or not).

The development of an unbiased, data-driven method to query existing data repositories would offer the opportunity to determine the characteristics, trends, and outcomes of LS without undue biases and preconceived notions. Furthermore, such an approach would serve as a framework to enable traditional high-throughput retrospective epidemiological analyses, real-time enterprise-wide surveillance activities, and provide a foundation for prospective observational studies. Thus, the aims of the current study were twofold: (1) Develop a method to identify a population of Service members with combat-related LE limb salvage and (2) describe the most prevalent injuries and procedures experienced by that population.

## 2. Methods

### 2.1. Definition of Study Populations

**Figure 1:**
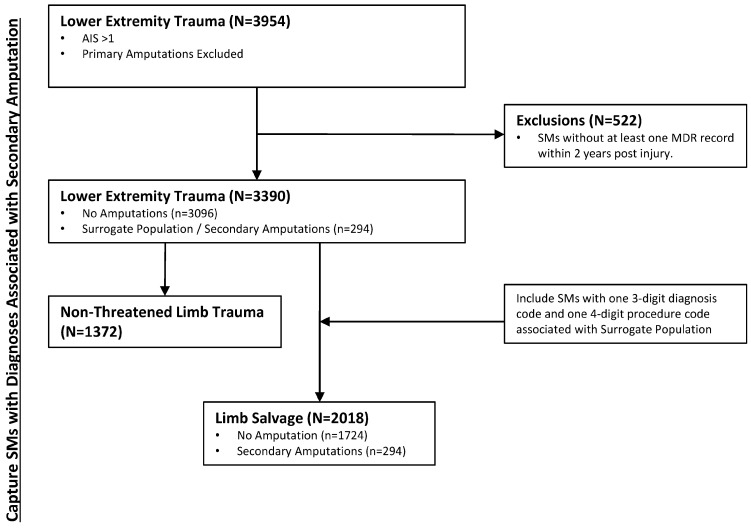
Graphical representation of the various sub-groups witin the population of Service members with combat-related extremity trauma, including those which are characterized as limb salvage.

This study was approved by the Naval Health Research Center (NHRC) Institutional Review Board; all methods were performed in accordance with the relevant guidelines and regulations. Utilizing the approved waiver of informed consent, a retrospective database review of all combat-related injuries to lower extremities from 2002 to 2014 with an acute injury episode documented in the Expeditionary Medical Encounter Database (EMED; NHRC, San Diego, CA, USA) [17] was performed. Within the EMED, there were 3954 SMs identified with a combat-related lower extremity (LE) injury not including those who underwent a primary amputation. Primary amputations were defined herein as any amputation, traumatic or surgical, occurring with 15 days of injury. This 15-day line of demarcation between primary and secondary amputations was chosen to account for the differences in treatment timelines between civilian and combat-related trauma. While civilian guidelines would suggest amputation as a primary course of clinical treatment to occur within 72 h of injury, timelines for combat-related injuries are challenged by the resourcing limitation of far-forward medical units and the subsequent need to transport the casualty to higher echelons of care where proper assessment of the injuries can made. As such, to avoid biasing our population with SMs carrying unsalvageable injuries we established a conservative 15-day threshold for qualification as a secondary amputation and excluded all others. From our initial sample, we excluded individuals with a maximum Lower Extremity Abbreviated Injury Scale (AIS) score of one (i.e., minor trauma) from further analysis. Previous research has demonstrated that this level of injury is not associated with secondary amputation [18]. On the other hand, we intentionally included patients with moderate injuries (i.e., AIS = 2) in our subject pool. This deliberate inclusion stems from the fact that the differentiation between a limb that requires salvage and one that is not acutely threatened can be unclear, and our overarching goal is to develop a method capable of objectively distinguishing between these two populations in a retrospective data set. Additional inclusion criteria included the availability of inpatient medical records within two years of the date of injury accessible within the Military Health System Data Repository (MDR). Within the LE trauma (AIS > 1) study population (n = 3390), a subpopulation of SMs who underwent secondary major LE amputation (i.e., partial foot and proximal) was identified and designated as the surrogate population (i.e., limb salvage case that proceeded to amputation) on which the data-driven LS definition approach would be based. All other members of the LE trauma population that were not identified as part of the limb salvage population were grouped as a non-threatened limb trauma population (Figure 1).

### 2.2. Systematic Grouping of Medical Codes for Discriminant Analysis

Diagnosis and inpatient procedures of the study population were determined from the EMED and the MDR, respectively, using International Classification of Diseases, 9th Revision, Clinical Modification (ICD-9 CM) diagnosis and procedure codes. Given the hierarchical nature of the ICD coding structure, a tradeoff exists between the specificity of the diagnosis or procedure codes and the predictive power of discriminating LS populations from less severe cases. To evaluate the impact of this issue in a systematic manner, a sensitivity analysis was performed on the number of digits, which correspond to levels of hierarchy, to be included in the data-driven LS classification. Using a significant association as a criterion for inclusion in the data-driven classification of LS, sensitivity, specificity, positive predictive value (PPV), and negative predictive value (NPV) of the determination of secondary amputation was calculated for each level of the diagnosis and procedure codes (Table 1). A minimum acceptable sensitivity of 90% and/or maximizing specificity was established as a decision criterion for establishing the number of digits to be included in the LS definition. Subsequently, diagnosis codes were grouped into three-digit families and procedure codes were evaluated using their full expression (i.e., four digits) for further analysis.

### 2.3. Determination of Medical Codes Associated with Limb Salvage

The frequency and percentages of initial injury diagnosis code families and inpatient procedure codes were calculated for the LE Trauma (AIS > 1) study population, and comparisons were made between the surrogate population (i.e., secondary amputation) and those SMs without limb loss to identify medical codes that are significantly associated with the LS (Table 2). Statistical significance was determined using a chi-square test, or Fisher’s exact test where appropriate, with a Bonferroni correction for multiple comparisons. Subsequently, an “OR” gating strategy was used to join significantly associated ICD-9 codes into a set of diagnoses and procedures, respectively. These sets were then joined through an “AND” gating strategy to arrive at a population ostensibly consisting of all SMs who underwent LE LS regardless of whether the limb was ultimately retained (Table 3). To put it another way, an SM was included in the LS population if and only if they were associated with both a diagnosis and a procedure that was significantly associated with the surrogate population. All procedures during the 2-year follow-up period were considered for inclusion in this definition.

### 2.4. Validation of Data-Driven Limb Salvage Definition Approach

The data-driven LS definition, and resultant cohort population, was validated against the consensus opinion of a panel of subject matter experts (SME) consisting of five experienced military trauma surgeons with backgrounds in both orthopedic surgery and plastic and reconstructive surgery. The SME panel was asked to review the diagnosis and procedure codes of a randomly selected sample of 50 SMs from the initial LE trauma study population. Upon completion of the review, each member of the SME panel was asked to classify each case as either LS or not LS. The SME panel was blinded to all the following: all other aspects of the subjects’ medical records besides the diagnosis and procedure codes, the decisions of other panel members, and the data-driven classification for all cases. The majority decision (i.e., ≥3/5) was used when there was disagreement across raters on the determination of an LS versus non-LS case. A percent agreement was calculated and the Fleiss methodology was utilized for Kappa statistics due to multiple raters.

## 3. Results

### 3.1. Diagnoses Significantly Associated with Limb Salvage

In total, 137 three-digit diagnosis code families were examined for the cohort of SMs with LE trauma. Of these diagnosis families, tibia and fibula fracture (823), metatarsal/tarsal fracture (825), lower extremity blood vessel injury (904), ankle fracture (824), and foot or ankle dislocation (838, 837) were significantly associated with the surrogate LS population (Table 2). Of the significantly associated diagnoses, tibia/fibula fracture was the most prevalent occurring in 37.91% of all LE trauma cases, with 60.88% of cases within the surrogate population having this diagnosis compared to 35.72% of the cases without an amputation. Metatarsal/tarsal fracture was also highly prevalent in the study population (27.79% of all cases) and was even more polarized in terms of distribution among subgroups with 60.88% of the surrogate population having that diagnosis relative to 24.64% cases not included in the surrogate population. While foot and ankle dislocations were not highly prevalent overall (5.66% and 3.66%, respectively), they were also highly polarized with their occurrence being more frequent in the surrogate population (i.e., 3–4 times more prevalent). Other highly prevalent diagnoses (n = 21) within the population that were not found to be significantly associated with LS are included in Supplemental Table S1. Among these non-significantly associated diagnoses are femur fractures (821), lower extremity nerve injuries (956), various open wounds affecting the extremity, and injuries associated with polytrauma (e.g., concussion, head injuries).

### 3.2. Procedures Significantly Associated with Limb Salvage

A sum of 314 procedure codes were examined for significant association with LS. Of the codes examined, 68 were found to be significantly associated with our surrogate population (Table 3 and Supplemental Table S2). Of the significantly associated procedures, the non-excisional debridement of wound, infection, or burn (86.28) was the most frequent (71.1%; *p* ≤ 0.00001 secondary amputation vs. no amputation) procedure for SMs within the surrogate population as well as in the broader LE trauma population (52.6%). ICD-9 codes 86.59 (closure of skin and subcutaneous tissue of other sites), 86.22 (excisional debridement of wound, infection, or burn), and 99.04 (transfusion of packed cells) were documented in more than half of the surrogate population (57.1%, 60.4%, and 61.6%, respectively). Other procedure codes such as 77.67 (local excision of lesion or tissue of bone), 78.47 (other repair or plastic operation on bone), 84.72 (application of external fixator device), and 78.67 (removal of implanted devices from bone) were documented more frequently in the surrogate population than the no amputation or broader LE trauma groups. The 25 most frequent significantly associated procedures among LS are listed in Table 3. All other significantly associated procedures are found in Supplementary Table S2.

### 3.3. Prevalence of Limb Salvage Cases in Combat-Related Extremity Trauma

The conjunction of the set of diagnosis (Table 2) and procedure codes (Table 3 and Table S2) was used to define a population of n = 2018 SM who underwent LS. This population, therefore, represents 59.5% of the initial study population with LE trauma. The remaining 40.5% (n = 1372) represents the subset of the population that experienced LE trauma that could be classified as non-limb threatening.

Among the LS cohort, the most prevalent diagnoses were fractures of the tibia/fibula (59.6% of LS, PPV: 93.6), metatarsal/tarsal (42.2% of LS, PPV: 90.3), and vascular injury to the lower extremity (24.4% of LS, PPV: 94.8) (Table 4) which were mostly in line with the surrogate population that underwent secondary amputation. Metatarsal/tarsal fractures represented an exception and were slightly lower (60.9% vs. 42.2%) in the broader LS population relative to the secondary amputation cohort on which its defining parameters were derived. Accordingly, metatarsal/tarsal fracture had the lowest PPV for identifying LS cases of all the significantly associated diagnoses. Ankle and foot dislocations, the least prevalent of the significantly associated diagnoses, exhibited the highest PPV and lowest NPV for LS.

The most prevalent procedures among the LS cohort pertained to non-excisional (58.8% of LS, PPV: 66.3) and excisional (47.2% of LS, PPV: 69.0) debridement and skin closure (48.8%, PPV: 67.7) (Table 5). Given the ubiquitous nature of these procedures, it is unsurprising that the PPV of these procedure codes is relatively low. Conversely, the procedure codes with the highest PPV for discriminating LS from other extremity traumas were not highly prevalent; specifically, these procedures related to the open reduction of metatarsal/tarsal (14.7% of LS, PPV: 99.7) and tibia/fibula fractures (26.8% of LS, PPV: 97.5) with internal fixation, application of external fixators for tibia/fibula fractures (13.8% of LS, PPV: 99.6), the removal of implants from the tibia/fibula (16.1% of LS, PPV: 98.2), and debridement of open fracture sites affecting the tarsal/metatarsal (9.1% of LS, PPV: 99.5), and tibia/fibula (25.5% of LS, PPV: 96.3).

### 3.4. Validation of Data-Driven Approach for Defining Limb Salvage

The data-driven approach for classifying LS cases was validated against the gold standard approach of SME review/opinion. Of the 50 test cases, 35 (70%) were found to be in agreement between the two classifications (i.e., data-driven classification and SME classification) (Table 6). The Kappa statistic (κ = 0.55) indicates a moderate agreement between the data-driven approach and the expert opinion of the SME panel. Of the 15 discrepancies, three cases were categorized as “no” for LS according to the data-driven classification but “yes” according to the majority SME classification. On the other hand, 12 of the cases were categorized as “yes” according to the data-driven classification but “no” based on the majority SME classification. The sensitivity and specificity were identified as 55.6% (expert range of 51.8–66.7%) and 87% (expert range of 73.9–91.3%), respectively.

## 4. Discussion

### 4.1. Strengths

A recent report from a US Department of Defense International State-of-the-Science Meeting on Limb Salvage and Recovery after Blast-Related Injury highlighted the need for a definition of trauma-related limb salvage to be developed, validated, and published so as to enable research on the outcomes of limb salvage and relative efficacy of various surgical approaches [19]. Furthermore, it was suggested that such a definition be capable of evolving over time, be able to discriminate clinical care that is not LS, and that it should indicate that patients with amputations can also be considered patients with LS. In addition to these parameters, the influence of individual clinician/researcher biases should also be minimized. To this end, the approach described herein aimed to address these gap areas by creating and validating a data-driven method for identifying the historically ill-defined LS population. Through the analyses performed, it was determined that a specific subset of diagnosis and procedure code families associated with a surrogate population of cases that initially underwent LS but subsequently proceeded to undergo a secondary amputation could be used to create a data-driven algorithm which accurately identifies LS cases in an unbiased and high-throughput manner. The identified LS cohort represents 59.5% of the broader combat-related LE trauma population, and is most succinctly described as those cases with diagnoses of open fracture affecting the tibia/fibula and/or tarsal/metatarsals, and/or lower extremity vascular injuries and procedures associated with their surgical reconstruction, both general and particular in nature. The general procedures were both unsurprisingly frequent and poorly predictive of LS relative to the more particular procedures (i.e., open fracture debridement, reduction, and fixation) which were less frequent but highly predictive of LS.

Overall, the data-driven method to differentiate LE trauma cases as either LS or non-threated limb trauma exhibited moderate agreement (κ = 0.55) with the consensus opinion of an SME panel suggesting this approach is at least on par with standard practice in the literature. It is important to note that this level of agreement is in line with the observed inter-rater agreement of our SME panel (κ = 0.52).

### 4.2. Limitations

While the data-driven approach described herein represents a potentially powerful approach for studying the historically overlooked LS population, several decisions and key assumptions went into the development of this approach which leave room for further improvement. First and foremost, among these was the decision to use medical coding data as the basis for informing the LS inclusion/exclusion criteria. Clearly, the benefit of doing so is the ability to allow high throughput classification and to remove individual biases from the process. Unfortunately, however, medical coding errors do occur, and it is possible, even likely, that some diagnoses and procedures have been misidentified in the electronic health records. However, it is likely that these coding errors would generally be both minor in nature (e.g., within a code family) and uniformly distributed across all coding families [20]. As such, the effect of such errors on the results described herein are likely to be minimal, particularly given that codes were included in the LS based on hierarchical code families rather than individual codes, and uniformly distributed across the entire LE trauma population. Furthermore, it should be noted that ICD-9 codes were used in the development of this approach as it was the predominant coding strategy for the majority of the time period over which our data were captured. Given that ICD-9 has now been deprecated in lieu of the ICD-10 codes, application of this approach moving forward will require the conversion of the ICD-9 codes identified herein to their ICD-10 counterparts. Given ICD-10 is a more granular coding system, the mapping of ICD-9 to the ICD-10 codes is not bijective and thus will require careful conversion by a subject matter expert.

A second major limitation of this study is the decision to base the association of diagnosis and procedure codes to LS via univariate analysis. Multivariate analysis offers the opportunity to better control for potential confounders to the identified associations, including the mechanism and/or severity of injury as well as the correlation of procedures that may functionally be linked to LS procedures, but not necessarily characteristic of what one might intuitively think of as LS procedures. To illustrate how this limitation impacts our definition of LS, one can look to the areas of discordance between our data-driven approach and the SME panel from our validation study. In that analysis, there were 15 cases where discrepancies between the data-driven LS classification and the consensus of the SME panel occurred. These discrepancies could overwhelmingly be described as false positives (i.e., identification of cases as LS when, in fact, they are not) suggesting the specificity of the LS data-driven definition is too low. On the other hand, if a multivariate analysis had been used herein, the resulting LS definition may have been too restrictive inclusion/exclusion criteria and thus resulting in a narrower definition of limb salvage. Once again revisiting our validation study, we find that the three cases where the data-driven algorithm failed to identify cases that the SME panel determined to be LS revealed that those subjects presented with two injury patterns—femur fracture and lower extremity nerve injury—that often involve major reconstructive efforts and would seem to fit with an intuitive definition of LS. Given that our approach lacked the robustness to capture less frequent conditions (i.e., femur fracture, peripheral nerve injuries) within its criteria, the conservative decision was made to use a univariate approach so as to allow for a more inclusive definition rather than one that would be even more restrictive under multivariate analyses. As such, this introduces a somewhat systemic bias toward less severe cases being misclassified as LS, but importantly enhances the likelihood that true LS cases are captured.

The final limitation of our approach is that the quality of any data-driven classification system is inherently dependent on the size and characteristics of the surrogate population as well as the overall prevalence of specific diagnoses in the broader LE trauma population. To put it another way, if the surrogate population on which the definition of LS is derived is too small or the prevalence of the injury is underrepresented relative to the broader population, the resultant data-driven approach is unlikely to capture such diagnoses. In the present study, both possibilities likely contribute to the exclusion of the aforementioned diagnoses from the LS definition as both the overall study population (n = 3390) and surrogate population (n = 294) were relatively small on an epidemiological scale. With that said, it is crucial to emphasize the importance of replicating the current data-driven approach using other, potentially even larger databases. This replication process will play a pivotal role in refining the algorithm for diverse extremity trauma populations. This is because the characteristics of the combat-related limb salvage (LS) population are unlikely to closely align with those of the civilian trauma-related LS population. This divergence arises from disparities in age, physical fitness, and rehabilitation opportunities between civilian and military populations. With respect to rehabilitation opportunities, it is widely recognized that the US Military Health System offers more extensive rehabilitation opportunities compared to US civilian healthcare systems, which often grapple with challenges related to private insurance reimbursement [21,22]. Following a similar line of reasoning, it is conceivable that there is greater institutional support for attempting limb salvage procedures within the Military Health System than within civilian health systems, which might influence the characteristics of a military limb salvage cohort relative to a civilian cohort. Fortunately, this limitation is relatively easy to overcome as the approach outlined herein is easily translatable to other datasets so that such comparisons can be made by following the same methodology.

## 5. Conclusions

The goal of this study was to develop and validate a data-driven algorithm capable of identifying an LS population among SMs with combat-related LE trauma and subsequently describe the most prevalent diagnoses and procedures associated with their injuries. The described approach produced a definition of LS with relatively high specificity (87%) and moderate sensitivity (55.6%) that agreed favorably (κ = 0.55) with the consensus opinion of an SME panel. As such, the use of this definition can be utilized to enable future high-throughput retrospective analyses to capture trauma-related LS cases for studying the demographics, clinic utilization, complications, and ultimately long-term clinical outcomes of this underserved population of SM. Additionally, this tool could be utilized to pre-screen records for inclusion and/or serve as an objective quality control check of included records for LS studies for which manual extraction is planned or has been performed. Finally, these results could be used to establish a surveillance method to identify potential LS cases for recruitment into prospective studies.

## 6. Disclaimer

The views expressed in this abstract are those of the authors and do not reflect the official policy or position of the Department of Defense, Department of the Army, Department of the Navy, the Uniformed Services University of the Health Sciences, nor the United States Government.

## Figures and Tables

**Table 1:** Analysis of Predictive Value of Coding Strategies.

ICD-9 Code Grouping Strategy		Sensitivity	Specificity	Positive Predictive Value	Negative Predictive Value
**Diagnosis Codes**	**1-Digit Families**	71.43%	36.34%	9.63%	93.05%
	**2-Digit Families**	95.58%	21.64%	10.38%	98.10%
	**3-Digit Families**	91.84%	36.79%	12.12%	97.94%
	**4-Digit Families**	84.69%	62.08%	17.50%	97.71%
**Procedure Codes**	**1-Digit Families**	99.32%	7.01%	9.21%	99.09%
	**2-Digit Families**	99.32%	8.59%	9.35%	99.25%
	**3-Digit Families**	98.64%	11.37%	9.56%	98.88%
	**4-Digit Families**	98.64%	12.86%	9.71%	99.00%

**Table 2:** Identification of ICD Diagnosis Codes Associated with our Surrogate Population.

ICD-9 Code	Description	Frequency (*f*)	Percentage (%)	LE Trauma (AIS > 1) N = 3390				PPV	NPV	*p*-Value
				No Amputation n = 3096		Surrogate Population n = 294				
				*f*	%	*f*	%			
**823**	tib/fib fx	1285	37.91	1106	35.72	179	60.88	13.9	94.5	<0.0001
**825**	metatarsal/tarsal fx	942	27.79	763	24.64	179	60.88	19.0	95.3	<0.0001
**904**	LE blood vessel injury	520	15.34	435	14.05	85	28.91	16.3	92.7	<0.0001
**824**	fx ankle (tib/fib)	488	14.40	404	13.05	84	28.57	17.2	92.8	<0.0001
**838**	foot dislocation	192	5.66	118	3.82	74	25.17	38.5	93.1	<0.0001
**837**	ankle dislocation	124	3.66	86	2.78	38	12.93	30.6	92.2	<0.0001

**Note**: Only diagnosis code families that are significantly associated with our surrogate population (i.e., secondary amputation) after Bonferroni correction. Descriptive statistics for remaining diagnosis codes are provide in Supplemental Table S1.

**Table 3:** Identification of Procedure Codes Associated with our Surrogate Population.

ICD-9 Code	Description	LE Trauma (AIS > 1) N = 3390						PPV	NPV	*p*-Value
		Frequency (*f*)	Percentage (%)	No Amputation n = 3096		Surrogate Population n = 294				
				*f*	%	*f*	%			
**86.28**	Nonexcisional debridement of wound, infection, or burn	1782	52.6	1573	50.8	209	71.1	11.7	94.7	<0.00001
**86.59**	Closure of skin and subcutaneous tissue of other sites	1454	42.9	1286	41.5	168	57.1	11.6	93.5	<0.00001
**86.22**	Excisional debridement of wound, infection, or burn	1392	41.1	1214	39.2	178	60.5	12.8	94.2	<0.00001
**99.04**	Transfusion of packed cells	1058	31.2	877	28.3	181	61.6	17.1	95.2	<0.00001
**86.69**	Other skin graft to other sites	738	21.8	617	19.9	121	41.2	16.4	93.5	<0.00001
**88.38**	Other computerized axial tomography	713	21.0	623	20.1	90	30.6	12.6	92.4	0.000025
**96.59**	Other irrigation of wound	658	19.4	576	18.6	82	27.9	12.5	92.2	0.00012
**38.93**	Venous catheterization, not elsewhere classified	595	17.5	482	15.6	113	38.4	19.0	93.5	<0.00001
**79.36**	Open reduction of fracture with internal fixation(tib/fib)	555	16.4	470	15.2	85	28.9	15.3	92.6	<0.00001
**79.66**	Debridement of open fracture site (tib/fib)	535	15.8	441	14.2	94	32.0	17.6	93.0	<0.00001
**83.45**	Other myectomy	474	14.0	378	12.2	96	32.6	20.3	93.2	<0.00001
**93.59**	Other immobilization, pressure, and attention to wound	472	13.9	402	13.0	70	23.8	14.8	92.3	<0.00001
**93.57**	Application of other wound dressing	438	12.9	375	12.1	63	21.4	14.4	92.2	<0.00001
**99.21**	Injection of antibiotic	380	11.2	294	9.5	87	29.6	22.8	93.1	<0.00001
**78.67**	Removal of implanted devices from bone (tib/fib)	331	9.8	249	8.0	82	27.9	24.8	93.1	<0.00001
**79.37**	Open reduction of fracture with internal fixation (tarsal/MT)	297	8.8	228	7.4	69	23.5	23.2	92.7	<0.00001
**78.17**	Application of external fixator device (tib/fib)	279	8.2	204	6.6	75	25.5	26.9	93.0	<0.00001
**93.56**	Application of pressure dressing	256	7.5	215	6.9	41	13.9	16.0	91.9	0.000014
**93.54**	Application of splint	225	6.6	190	6.1	35	11.9	15.6	91.8	0.00015
**99.99**	Other	220	6.5	183	5.9	37	12.6	16.8	91.9	<0.00001
**86.04**	Other incision with drainage of skin and subcutaneous tissue	213	6.3	170	5.5	43	14.6	20.2	92.1	<0.00001
**79.67**	Debridement of open fracture site (tarsal/MT)	185	5.5	129	4.2	56	19.0	30.3	92.6	<0.00001
**88.48**	Arteriography of femoral and other lower extremity arteries	180	5.3	139	4.5	41	13.9	22.8	92.1	<0.00001
**99.07**	Transfusion of other serum	166	4.9	129	4.2	37	12.6	22.3	92.0	<0.00001
**04.81**	Injection of anesthetic into peripheral nerve for analgesia	160	4.7	128	4.1	32	10.9	20.0	91.9	<0.00001

**Note:** For illustrative purposes, the 25 most frequent procedure codes that are significantly associated with our surrogate population (i.e., secondary amputation) after Bonferroni Correction are presented. Descriptive statistics for remaining significantly associated codes are provided in Supplemental Table S2.

**Table 4:** Descriptive Statistics and Predictive Value of Diagnoses for Limb Salvage Population.

ICD-9 Code	Description	Frequency *(f)*	Percentage (%)	LE Trauma (AIS > 1) N = 3390				PPV	NPV	*p*-Value
				Limb Salvage n = 2018		Non-Threatened Limb Trauma n = 1372				
				*f*	%	*f*	%			
**823**	tib/fib fx	1285	37.91	1203	59.6	82	6.0	93.6	61.3	<0.0001
**825**	metatarsal/tarsal fx	942	27.79	851	42.2	91	6.6	90.3	52.3	<0.0001
**904**	blood vessel injury LE	520	15.34	493	24.4	27	2.0	94.8	46.9	<0.0001
**824**	fx ankle (tib/fib)	488	14.40	444	22.0	44	3.2	91.0	45.8	<0.0001
**838**	foot dislocation	192	5.66	185	9.2	7	0.5	96.3	42.7	<0.0001
**837**	ankle dislocation	124	3.66	121	6.0	3	0.2	97.6	41.9	<0.0001

**Table 5:** Descriptive Statistics and Predictive Value of Procedures for Limb Salvage Population.

ICD-9 Code	Description	Frequency (*f*)	Percentage (%)	LE Trauma (AIS > 1) N = 3390				PPV	NPV	*p*-Value
				Limb Salvage n = 2018		Non-Threatened Limb Trauma n = 1372				
				*f*	%	*f*	%			
**86.28**	Nonexcisional debridement of wound, infection, or burn	1782	52.6	1181	58.5	601	43.8	66.3	47.9	<0.00001
**86.59**	Closure of skin and subcutaneous tissue of other sites	1454	42.9	985	48.8	469	34.2	67.7	46.6	<0.00001
**86.22**	Excisional debridement of wound, infection, or burn	1392	41.1	961	47.2	431	31.4	69.0	47.1	<0.00001
**99.04**	Transfusion of packed cells	1058	31.2	758	37.6	300	21.9	71.6	46.0	<0.00001
**86.69**	Other skin graft to other sites	738	21.8	538	26.7	200	14.6	72.9	44.2	<0.00001
**88.38**	Other computerized axial tomography	713	21.0	501	24.8	212	15.4	70.3	43.3	<0.00001
**96.59**	Other irrigation of wound	658	19.4	444	22.0	214	15.6	67.5	42.4	<0.00001
**38.93**	Venous catheterization, not elsewhere classified	595	17.5	410	20.3	185	13.5	68.9	42.5	<0.00001
**79.36**	Open reduction of fracture with internal fixation (tib/fib)	555	16.4	541	26.8	14	1.0	97.5	47.9	<0.00001
**79.66**	Debridement of open fracture site (tib/fib)	535	15.8	515	25.5	20	1.5	96.3	47.4	<0.00001
**83.45**	Other myectomy	474	14.0	340	16.8	134	9.8	71.7	42.5	<0.00001
**93.59**	Other immobilization, pressure, and attention to wound	472	13.9	335	16.6	137	10.0	71.0	42.3	<0.00001
**93.57**	Application of other wound dressing	438	12.9	298	14.8	140	10.2	68.0	41.7	0.0001
**99.21**	Injection of antibiotic	380	11.2	291	14.4	89	6.5	76.6	42.6	<0.00001
**78.67**	Removal of implanted devices from bone (tib/fib)	331	9.8	325	16.1	6	0.4	98.2	44.7	<0.00001
**79.37**	Open reduction of fracture with internal fixation (tarsal/MT)	297	8.8	296	14.7	1	<0.1	99.7	44.3	<0.00001
**78.17**	Application of external fixator device (tib/fib)	279	8.2	278	13.8	1	<0.1	99.6	44.1	<0.00001
**93.56**	Application of pressure dressing	256	7.5	172	8.5	84	6.1	67.2	41.1	0.009
**93.54**	Application of splint	225	6.6	193	9.6	32	2.3	85.8	42.3	<0.00001
**99.99**	Other	220	6.5	141	7.0	79	4.8	64.1	40.8	NS
**86.04**	Other incision with drainage of skin and subcutaneous tissue	213	6.3	158	7.8	55	4.0	74.2	41.4	<0.00001
**79.67**	Debridement of open fracture site (tarsal/MT)	185	5.5	184	9.1	1	<0.1	99.5	42.8	<0.00001
**88.48**	Arteriography of femoral and other lower extremity arteries	180	5.3	152	7.5	28	2.0	84.4	41.9	<0.00001
**99.07**	Transfusion of other serum	166	4.9	107	5.3	59	4.3	64.5	40.7	NS
**04.81**	Injection of anesthetic into peripheral nerve for analgesia	160	4.7	117	5.8	43	3.1	73.1	41.1	0.0003

**Table 6:** Validation of LS Cohort Against Consensus SME Opinion.

Limb Salvage?		SME Classification	
		Yes	No
**Data-Driven** **Classification**	Yes	n = 15	n = 12
	No	n = 3	n = 20

## Data Availability

All data supporting the findings of this study are available within the paper and its Supplementary Information.

## Supplementary Materials

The following supporting information can be downloaded at: https://www.mdpi.com/article/10.3390/jcm12196357/s1, Table S1: Descriptive statistics of Highly Prevalent ICD-9 Codes Not Associated with Limb Salvage; Table S2: Additional ICD-9 Procedure Codes Associated with the Surrogate Population.
