# Supplementary material for: A Data-Driven Method to Discriminate Limb Salvage from Other Combat-Related Extremity Trauma
Source: J Clin Med. 2023 Oct 4;12(19):6357. doi: 10.3390/jcm12196357 (PMC10573244; doi:10.3390/jcm12196357)
Supplement: Supplementary file 1 [file jcm-12-06357-s001.zip › jcm-2609144-supplementary.pdf]

Table S1: Descriptive statistics of Highly Prevalent ICD-9 Codes Not Associated with Limb Salvage

ICD-9 Code	Description	Frequency (*f*)	Percentage (%)	LE Trauma (AIS>1) N=3390				PPV	NPV	p-value
				No Amputation n=3096		Surrogate Population n=294				
				f	%	*f*	%			
**891**	*open wound knee, leg ankle*	1195	35.25	1090	35.21	105	35.71	8.8	91.4	0.86
**890**	*open wound hip, thigh*	1057	31.18	983	31.75	74	25.17	7.0	90.6	0.02
**850**	*concussion*	914	26.96	827	26.71	87	29.59	9.5	91.6	0.29
**873**	*open head wound*	849	25.04	776	25.06	73	24.83	8.6	91.3	0.93
**821**	*femur fx (shaft/distal end)*	536	15.81	470	15.18	66	22.45	12.3	92.0	0.001
**881**	*open wound elbow, forearm, wrist*	522	15.40	479	15.47	43	14.63	8.2	91.2	0.70
**956**	*nerve injury LE*	472	13.92	414	13.37	58	19.73	12.3	91.9	0.003
**805**	*fx vert. column no SCI*	463	13.66	412	13.31	51	17.30	11.0	91.7	0.05
**880**	*open wound shoulder/upper arm*	428	12.63	407	13.15	21	7.14	4.9	90.8	0.003
**872**	*open wound ear*	364	10.74	331	10.69	33	11.22	9.1	91.4	0.78
**808**	*pelvis fx*	339	10.00	314	10.14	25	8.50	7.4	91.2	0.37
**910**	*superficial face, neck*	337	9.94	311	10.05	26	8.84	7.7	91.2	0.51
**877**	*open wound buttock*	328	9.68	299	9.66	29	9.86	8.8	91.3	0.91
**959**	*other injury unspecified*	306	9.03	281	8.99	25	8.50	8.2	91.3	0.74
**916**	*superficial LE*	305	9.00	287	9.27	18	6.12	5.9	91.1	0.07
**813**	*fx radius/ulna*	287	8.47	259	8.37	28	9.52	9.8	91.4	0.49
**802**	*face bone fx*	283	8.35	251	8.11	30	10.20	10.7	91.5	0.21
**882**	*open wound hand*	283	8.35	262	8.46	21	7.14	7.4	91.2	0.43
**879**	*open wound not specified except limbs*	281	8.29	261	8.43	20	6.80	7.1	91.2	0.33
**948**	*burns classified according to extent of body surface involved*	268	7.91	244	7.88	24	8.16	9.0	91.4	0.86
**892**	*open wound of foot except toe(s) alone*	254	7.49	224	7.24	30	10.20	11.8	91.6	0.06

Table S2: Additional ICD-9 Procedure Codes Associated with the Surrogate Population

ICD-9 Code	Description	Frequency (*f*)	Percentage (%)	LE Trauma (AIS>1) N=3390: No Amputation n=3096 *f*	No Amputation %	Surrogate Population n=294 *f*	Surrogate Population %	PPV	NPV	p-value
**78.07**	Bone graft (tib/fib)	154	4.5	122	3.9	32	10.9	20.8	91.9	<0.00001
**83.14**	Fasciotomy	152	4.5	118	3.8	34	11.6	22.4	92.0	<0.00001
**93.39**	Other physical therapy	145	4.3	113	3.6	32	10.9	22.1	91.9	<0.00001
**78.57**	Internal fixation of bone without fracture reduction (tib/fib)	144	4.2	119	3.8	25	8.5	17.4	91.7	0.00015
**86.74**	Attachment of pedicle or flap graft to other sites	143	4.2	114	3.7	29	9.9	20.3	91.8	<0.00001
**86.67**	Dermal regenerative graft	143	4.2	110	3.5	33	11.2	23.1	92.0	<0.00001
**93.83**	Occupational therapy	133	3.9	99	3.2	34	11.6	25.6	92.0	<0.00001
**84.52**	Insertion of recombinant bone morphogenetic protein	121	3.6	93	3.0	28	9.5	23.1	91.9	<0.00001
**84.72**	Application of external fixator device, ring system	120	3.5	80	2.6	40	13.6	33.3	92.2	<0.00001
**38.70**	Interruption of the vena cava	116	3.4	94	3.0	22	7.5	19.0	91.7	0.00006
**78.47**	Other repair or plastic operations on bone (tib/fib)	114	3.4	78	2.5	36	12.2	31.6	92.1	<0.00001
**78.69**	Removal of implanted devices from bone (other)	108	3.2	84	2.7	24	8.2	22.2	91.8	<0.00001
**83.82**	Graft of muscle or fascia	104	3.1	83	2.7	21	7.1	20.2	91.7	0.000022
**86.09**	Other incision of skin and subcutaneous tissue	99	2.9	79	2.5	20	6.8	20.2	91.7	0.00003
**77.67**	Local excision of lesion or tissue of bone (Tib/Fib)	99	2.9	67	2.2	32	10.9	32.3	92.0	<0.00001
**99.05**	Transfusion of platelets	99	2.9	70	2.3	29	9.9	29.3	91.9	<0.00001
**79.69**	Debridement of open fracture site (other specified bone)	97	2.9	77	2.5	20	6.8	20.6	91.7	0.00002
**83.32**	Excision of lesion of muscle	90	2.6	66	2.1	24	8.2	26.7	91.8	<0.00001
**83.39**	Excision of lesion of other soft tissue	86	2.5	62	2.0	24	8.2	27.9	91.8	<0.00001
**79.17**	Closed reduction of fracture with internal fixation (tarsal/MT))	84	2.4	64	2.0	20	6.8	23.8	91.7	<0.00001
**78.15**	Application of external fixator device (femur)	76	2.2	57	1.8	19	6.5	25.0	91.7	<0.00001
**97.14**	Replacement of other device for musculoskeletal immobilization	74	2.2	57	1.8	17	5.8	23.0	91.6	<0.00001
**86.70**	Pedicle or flap graft, not otherwise specified	63	1.9	44	1.4	19	6.5	30.2	91.7	<0.00001
**78.68**	Removal of implanted devices from bone (tarsal/MT)	63	1.9	39	1.3	24	8.2	38.1	91.9	<0.00001
**78.18**	Application of external fixator device (tarsals/MT)	62	1.8	39	1.3	23	7.8	37.1	91.9	<0.00001
**81.13**	Subtalar fusion	59	1.7	43	1.4	16	5.4	27.1	91.7	<0.00001

**86.75**	Revision of pedicle or flap graft	51	1.5	39	1.3	12	4.1	23.5	91.6	0.00014
**78.19**	Application of external fixator device (other)	51	1.5	34	1.1	17	5.8	33.3	91.7	<0.00001
**84.51**	Insertion of interbody spinal fusion device	50	1.5	38	1.2	12	4.1	24.0	91.6	0.00010
**93.44**	Other skeletal traction	47	1.4	32	1.0	15	5.1	31.9	91.7	<0.00001
**77.69**	Local excision of lesion or tissue of bone (other)	42	1.2	31	1.0	11	3.7	26.2	91.5	0.00005
**78.58**	Internal fixation of bone without fracture reduction (tarsal/MT)	39	1.1	25	0.8	14	4.8	35.9	91.6	<0.00001
**79.07**	Closed reduction of fracture without internal fixation (tarsal/MT)	35	1.0	24	0.8	11	3.7	31.4	91.6	<0.00001
**83.85**	Other change in muscle or tendon length	34	1.0	24	0.8	10	3.4	29.4	91.5	0.000016
**81.11**	Ankle fusion	32	0.9	17	0.5	15	5.1	46.9	91.7	<0.00001
**93.53**	Application of other cast	31	0.9	22	0.7	9	3.1	29.0	91.5	0.00005
**77.68**	Local excision of lesion or tissue of bone (Tarsal/MT)	28	0.8	18	0.6	10	3.4	35.7	91.6	<0.00001
**93.24**	Training in use of prosthetic or orthotic device	21	0.6	12	0.4	9	3.1	42.9	91.5	<0.00001
**83.13**	Other tenotomy	20	0.6	12	0.4	8	2.7	40.0	91.5	<0.00001
**84.11**	Amputation of toe	16	0.5	8	0.2	8	2.7	50.0	91.5	<0.00001
**83.87**	Other plastic operations on muscle	13	0.4	7	0.2	6	2.0	46.2	91.5	<0.00001
**39.56**	Repair of blood vessel with tissue patch graft	13	0.4	6	0.2	7	2.4	53.8	91.5	<0.00001
**77.87**	Other partial ostectomy (tib/fib)	12	0.3	6	0.2	6	2.0	50.0	91.5	<0.00001
